# The role of ethnicity in pathways to emergency psychiatric services for clients with psychosis

**DOI:** 10.1186/s12888-017-1285-3

**Published:** 2017-04-13

**Authors:** Martin Rotenberg, Andrew Tuck, Rachel Ptashny, Kwame McKenzie

**Affiliations:** 1 University of Toronto, Centre for Addiction and Mental Health, Toronto, ON Canada; 2 Centre for Addiction and Mental Health, Toronto, ON Canada; 3 Centre for Addiction and Mental Health & Youthdale Treatment Centres, Toronto, ON Canada; 4 Social Aetiology of Mental Illness (SAMI) CIHR Strategic Training Initiative, Toronto, Canada

**Keywords:** Psychosis, Ethnicity, Pathways to care, Emergency department, Neighbourhood factors

## Abstract

**Background:**

This study investigates the role of ethnicity in pathways to emergency mental health care in Toronto for clients with psychosis, while taking into account neighborhood factors. Previous literature has focused on Afro-Caribbean clients, reporting an increased risk of accessing mental health care through negative pathways.

**Methods:**

A retrospective chart review for clients from 6 different ethnic origin groups presenting with psychosis – East Asian, South Asian, Black African, Black Caribbean, White European, and White North American – was undertaken in a psychiatric emergency department (ED). Logistic regression models were constructed to examine the relationship between pathways to care (involuntary detention under the Mental Health Act (MHA), police or ambulance referral, accompaniment by family or friends) with individual and neighbourhood factors.

**Results:**

A total of 765 clients were included in the study. East Asian (OR =2.36*, p* < 0.01) and South Asian (OR 2.99, *p* < 0.01) origin clients have increased odds of presenting to the ED while involuntarily detained under the MHA. Involuntary status under the MHA increased the odds of presenting via police or ambulance (OR 8.27, *p* < 0.001). East Asian origin clients have increased odds of presenting to the ED by police or ambulance (OR =2.10, *p <* 0.05). Clients from neighbourhoods with higher levels of residential instability have increased odds of presenting by police or ambulance (OR =1.35, *p <* 0.01), while clients from neighbourhoods with higher levels of ethnic concentration have increased odds of being accompanied to the ED by family or friends (OR =1.33, *p <* 0.01).

**Conclusion:**

In contrast to previous studies, East Asian and South Asian origin clients with psychosis have increased odds of a coercive pathway to emergency psychiatric services in Toronto. Black African and Black Caribbean origin clients do not have increased odds of a coercive pathway. Clients living in areas with high levels of residential instability are more likely to encounter a negative pathway. Ethnic concentration may be a supportive factor in family and friend accompaniment.

## Background

Improving mental health services so that they offer equitable outcomes for ethnic minority groups is a focus for policymakers in Canada [1]. With increasing immigration and diversity [2] healthcare systems have to be able to provide equitable services to ethnic minority populations.

There is a growing literature on differences in pathways to acute psychiatric care taken by clients with psychosis from different ethnicities [3–7]. Ethnic minority group members are more likely to access services through pathways that include police and ambulance referral and under involuntary detention through mental health legislation [3, 7]. These coercive experiences result in a loss of dignity and respect [8], lower levels of satisfaction with care [9, 10] and may be associated with poorer outcomes due to future reluctance to engage with the mental health care system, increased coercion and the need for involuntary detention at future interactions [7, 11]. If differences exist in the amount of coercion (e.g., frequency of involuntary detention under mental health legislation, police detainment and referral, use of community treatment orders) in the care pathways of specific ethnic groups it is important to understand why this is happening for a system to be able to offer equitable care. This is particularly important during times of crisis, when individuals may be more vulnerable and require emergency psychiatric services.

The majority of the literature investigating differences in care pathways has been conducted in the United Kingdom. A systematic review of 49 studies found all ethnic minority clients are more likely to be detained under mental health legislation when compared to White clients [4]. Much work has focused on differences between those of African-Caribbean origin and White clients. African-Caribbean clients have more complex pathways to specialized psychiatric care with greater police involvement and involuntary admissions [3]. The UK studies have led to recognition of the role of ethnicity and culture in service delivery and policy and is highlighted in evidence-based guidelines for the treatment and management of psychosis and schizophrenia published by the National Institute for Health and Care Excellence [12]. In the much smaller American literature, African American clients have been found to have more coercive referrals to psychiatric services with greater police involvement [13].

In Canada there is mixed evidence for differences in coercion and pathways to care for clients of different ethnic groups with psychosis. In the first study of its kind in Canada, Jarvis et al. reported that Montreal residents of Afro-Caribbean origin were more likely to be brought to hospital by police or ambulance [5]. In Ontario, pathways studies have focused on first episode clients with psychosis. One study reported increased odds of police involvement in the pathways of African and Caribbean origin clients when compared to White clients, and that those of Asian origin experience significantly less involuntary hospitalizations than both White and Black Canadians [14]. A recent first episode pathways study did not find significant differences in police involvement in the pathways of African, Caribbean and White clients [15].

Other clinical and demographic factors which may be linked to ethnicity are important in determining how clients access and engage with mental health services. Contact with a primary care provider may mitigate the odds of a negative pathway to care [16], however access and contact with primary care providers may differ between ethnic groups [17]. Specialized service delivery models like assertive community treatment may also play an important role in how services are utilized as they have been found to reduce hospitalization, decrease psychosocial stressors, and improve symptoms [18]. These outcomes may reduce the need for coercive measures during crisis, however, the use of community mental health services differs between ethnic groups [19] and ethnic minority group clients may face challenges secondary to cultural and linguistic needs in accessing specialized mental health services in the community, such as assertive community treatment teams [20].

In Ontario, the provincial Mental Health Act (MHA) outlines criteria for involuntary assessment and admission in specially designated psychiatric facilities, as well as the management of specific forms of outpatient care via community treatment orders (CTO). Under Section 17 of the MHA, police officers may detain and bring an individual into a hospital for assessment if there is “reasonable and probable grounds” that a person is acting in a “disorderly manner” or if the officer is of the opinion that the person is suffering from a mental disorder and there is a sufficient level of risk of harm [21]. These are all coercive measures that limit the rights of clients during times of crisis. This can be seen in contrast to a presentation that is voluntary and does not engage police and emergency medical services during the pathway to care, which can be perceived to be less coercive. From a clinical perspective, clients perceived to have more challenging presentations, including high risk behaviors and severe symptoms may be at increased risk for police involvement. It is important to consider the issue of racial stereotyping in relation to symptomatology and coercive measures, as young Black males may be perceived as more threatening, incoherent and disturbed in their presentation, which may contribute to increased coercion and police involvement [22].

Neighbourhood level socio-economic factors play a role in how clients access mental health services. Neighbourhood economic marginalization has been found to moderate ethnic disparities in accessing and using mental health services in the United States, with Black clients in high-poverty areas having increased rates of coercive referrals [13]. Using a semi-structured screening measure, ethnic minority group members are less likely to endorse that they have had psychotic experiences in the last year if they reside in areas with a larger proportion of own-group members [23]. In Ontario, clients with psychosis living in areas with a larger proportion of immigrants have higher rates of hospitalization [24].

To promote equity of outcomes it is important to identify disparities and improve pathways for all people with psychosis, not just first episode clients. However, to date, no study from Ontario (the province with the highest number of immigrants) has attempted to replicate Jarvis’s work [5] or investigate pathways to emergency psychiatric care for clients with psychosis in Canada in light of newer evidence of factors that may be of influence. This study aimed to investigate the mode of arrival to a mental health emergency department (ED) and the use of involuntary detention under the Ontario MHA for individuals with psychosis who identify as being a member of the largest ethnic minority groups in Toronto. We also sought to identify and compare socio-demographic, clinical, and neighborhood factors that predict involuntary presentation under the MHA, and factors considered to promote less coercive pathways such as being accompanied by friends and family as well as other factors known to influence pathways to care.

It is hypothesized that this study will reproduce findings that Black Caribbean and Black African clients with psychosis are more likely to present to the ED via a coercive pathway than White North American clients. Furthermore, it is hypothesized that neighbourhood factors will influence how clients present to the ED. Clients from neighbourhoods with higher levels of material deprivation will be more likely to experience a coercive pathway to care, while clients from neighbourhoods with higher levels of ethnic concentration will be less likely to experience a coercive pathway to care.

## Methods

### Study Design

A retrospective chart review was conducted of all clients attending a psychiatric ED in downtown Toronto between January 1, 2009 and December 31, 2011. All clients who came to the ED during this period were eligible for the study, however only their first ED visit was considered for inclusion.

To reduce the likelihood of a cohort effect with our sample we used a specific random selection process to identify potential participants. Clients were separated by first year of visit and a random number was generated for each client in each year. Clients were sorted by that random number for each year, and then the fourth record in each year was taken out and put into another file until four new files were created. The records in each file were worked through simultaneously by trained research volunteers and project team members until we reached 50 clients in each target ethnic group in 1 year or the end of the list of records was reached. This was done to purposefully reach a total sample size of 900 clients at the end of the 3-year period included in this study.

#### Inclusion Criteria

A three step method was used to select clients for inclusion. First, all clients receiving a chart diagnosis of schizophrenia, schizophreniform disorder, schizoaffective disorder, psychosis due to a substance or general medical condition, psychosis NOS (not otherwise specified), psychosis NYD (not yet diagnosed) or prodromal psychosis were selected. Second, from this group we aimed to select clients who identify as members of the 6 largest ethnic groups in Toronto based on the information in their chart:

i. East Asian (e.g., Chinese, Japanese, Korean);
ii. South Asian (e.g., Indian, Pakistani, Sri Lankan);
iii. Black African (e.g., Ghanaian, Kenyan, Somali);
iv. Black Caribbean (e.g., Barbadian, Jamaican);
v. White European (e.g., English, Italian, Portuguese, Russian); and
vi. White North American (e.g., Canadian, American).

Client ethno-racial data is self-defined and documented in a standardized assessment form based on categories developed by the Toronto District School board [25]. These groups are ethnic origin groups. We use the term “origin” to reflect the fact that many of our clients are either born in Canada or born in another country but are now Canadian citizens or residents.

Clients who self-identify as being a member of more than one ethnic origin group were excluded from this sample and had the option of selecting “mixed heritage” or “other”. Lastly, clients were only included if they had a mode of arrival documented in their chart.

#### Ethics

This study obtained approval from the research ethics board of the Centre for Addiction and Mental Health, Toronto, Ontario, Canada.

### Measures

#### Demographic Data

Basic demographic data including age at presentation coded as a continuous variable, gender (categorized as male or female) coded as a dichotomous variable, and relationship status (categorized as single or in a relationship) coded as a dichotomous variable were recorded for each client.

#### Health Care Providers

Data on whether clients have a treating psychiatrist or primary care physician in the community were coded as dichotomous variables.

#### Involuntary Status

Involuntary status under the Ontario MHA on arrival to the ED was determined by presentation to the ED under the following: Form 1 (application by physician for psychiatric assessment), Form 2 (order for examination by a justice of the peace), under police apprehension (Section 17 of the MHA), under court order (Sections 21–22 of the MHA), or a Form 47 (order for examination under a CTO). This was coded as a dichotomous variable.

#### Mode of Arrival

Negative mode of arrival was determined by presentation to the ED by police, ambulance, or a mobile crisis intervention team (police officer with specialized training and mental health nurse). This was coded as a dichotomous variable.

#### Family or Friend Accompaniment

Presentation to the ED with family or friend accompaniment was coded as a dichotomous variable.

#### Severity of Psychosis

An index of psychosis severity was constructed and coded as a continuous variable in a similar manner to previous studies assessing pathways to ED services [5] via data that was systematically collected during the chart review. 1 point was scored (up to a maximum of 14) for the presence of each of the following on mental status examinations and clinical history:

- hallucinations;
- delusions;
- ideas of reference;
- evidence of thought disorder;
- bizarre behavior;
- chart diagnosis of schizophrenia or schizoaffective disorder;
- previous psychiatric admission;
- evidence of self-neglect;
- guarded/suspicious/un-cooperative with clinical interview;
- poor insight;
- poor judgment;
- poor impulse control;
- aggressive behavior; and
- agitation.

#### Risk

A measure of risk was determined to be present if clients 1) presented to the ED to be assessed for harm to self, harm to others or a suicide attempt; or 2) if the presence of suicidal or homicidal thought content on mental status examination was noted. This was coded as a dichotomous variable.

#### Neighbourhood Marginalization

Clients who presented with a valid Ontario postal code were connected to a census dissemination area and linked to the Ontario Marginalization Index (ON-Marg), a validated census based index that provide data on neighbourhood level marginalization [26]. The four dimensions that make up this index and associated indicators are presented in Table 1. The dimensions are constructed via factor analysis of census data and have a standardized factor scale of deprivation with a mean of 0 and standard deviation of 1. Lower scores correspond to low marginalization and higher scores correspond to higher marginalization.

**Table 1:** Dimensions of marginalization and respective indicators of the ON-Marg

Dimension	Indicators
Residential instability	Proportion of the population living alone
	Proportion of the population aged 16+
	Average number of persons per dwelling
	Proportion of the population who are single/divorced/widowed
	Proportion of dwellings that are apartment buildings
	Proportion of dwellings that are not owned
	Proportion of the population who moved during the past 5 years
Material deprivation	Proportion of the population aged 20+ without a high-school diploma
	Proportion of families who are lone parents
	Proportion of the population receiving government transfer payments
	Proportion of the population aged 15+ who are unemployed
	Proportion of the population considered low-income
	Proportion of household living in dwellings that are in need of major repair
Dependency	Proportion of the population who are 65+
	Dependency ratio (total population aged 0–14 & 65+ / total population aged 15–64)
	Proportion of the population not participating in labour force (aged 15+)
Ethnic concentration	Proportion of the population who are recent immigrants
	Proportion of the population who self-identify as a visible minority

Adapted from the Ontario Marginalization Index User Guide Version 1.0

#### Statistical Analysis

All statistical analyses were conducted in SPSS 21.0 [27]. Chi-square tests were performed to examine the association between ethnicity and both involuntary status and mode of arrival. Binary logistic regression models were constructed to examine the relationship between the dichotomous outcome variables (involuntary status on presentation, negative mode of arrival, and accompaniment by family or friends) and predictors variables (demographic, clinical and neighbourhood factors). Predictor variables were included in the regression models only if there was an association or difference with the outcome variable via bivariate analyses with a liberal *p*-value of ≤0.10 or reported in previous literature. Hierarchical regression models were constructed in which demographic and clinical predictor variables were utilized in the first model, and then in subsequent models neighbourhood level variables were added. Individuals who did not have a valid postal code, and therefore did not have neighbourhood level marginalization data, were excluded from models that included neighbourhood level variables. One model with mode of arrival as an outcome was constructed in a similar manner to replicate the methodology utilized in a previous study (5) before additional variables that were associated with the outcome measure were added.

## Results

In total 9764 unique client records were examined. Of these, 5776 clients were excluded because they did not meet the inclusion criteria for diagnosis. A further 560 clients were excluded as they did not identify with one of the six target ethnic origin groups and 114 clients were excluded because there was either no mode of arrival, or multiple modes documented. Another 1274 clients were excluded due to a combination of the above criteria. Due to privacy restrictions on the charts of 4 clients they were not eligible for review and excluded. There were 400 blank forms, and 871 clients were excluded because we reached our participant cap of 50 for any ethnic group in 1 year. A total of 765 clients meeting all inclusion criteria were selected for analysis in this study (see Table 2 for the sample description).

**Table 2:** Descriptive profile of emergency department clients with diagnosis of psychosis by ethnic group origin (*n* = 765)

Variable	East Asian (*n* = 137)		South Asian (*n* = 91)		Black African (*n* = 99)		Black Caribbean (*n* = 141)		White North American (*n* = 150)		White European (*n* = 147)				
	*n*	%	*n*	%	*n*	%	*n*	%	*n*	%	*n*	%	Test statistic	df	*p*
Age (Mean ± SD)	37.8 ± 14.9		35.7 ± 11.9		33.5 ± 11.1		39.2 ± 14.0		39.8 ± 13.8		39.7 ± 16.1		F = 3.68	5, 754	0.003
Female	63	46.7	29	32.2	35	35.4	57	41.6	45	30.8	46	32.2	Χ^2^ = 11.49	5	0.043
Partner	17	13.5	13	15.9	6	6.8	9	6.9	19	13.8	14	10.0			ns
GP	69	51.5	46	51.1	40	41.2	64	46.4	65	44.2	74	51.4			ns
Psychiatrist	79	59.4	45	50	60	61.9	73	52.5	87	59.2	82	56.6			ns
Symptom Severity Scale (Mean ± SD)	4.5 ± 2.3		4.4 ± 2.4		4.9 ± 2.5		5.5 ± 2.8		5.0 ± 2.6		4.6 ± 2.5		F = 3.23	5, 759	0.007
Arrival by Police or Emergency	54	39.4	31	34.1	38	38.4	52	36.9	43	28.7	52	35.4			ns
Accompanied by Friend or Family	33	24.1	18	19.8	22	22.2	24	17.0	33	22.0	41	27.9			ns
Involuntary Mental Health Status	47	35.6	34	38.2	35	36.5	50	35.7	36	25.2	44	30.6			ns
Clinical Risk	42	30.7	27	29.7	25	25.3	37	26.2	41	27.3	46	31.3			ns
ON-Marg	East Asian (*n* = 109)		South Asian (*n* = 69)		Black African (*n* = 81)		Black Caribbean (*n* = 108)		White North American (*n* = 114)		White European (*n* = 125)		Test statistic	df	*p*
Residential Instability (Mean ± SD)	0.99 ± 1.28		0.75 ± 1.23		1.07 ± 1.33		1.10 ± 1.18		1.00 ± 1.22		0.94 ± 1.18				ns
Material Deprivation (Mean ± SD)	0.19 ± 1.11		0.16 ± 1.15		0.68 ± 1.53		0.82 ± 1.56		0.06 ± 1.12		0.16 ± 1.02		F = 10.40	5, 600	<0.001
Dependency (Mean ± SD)	−0.23 ± 0.75		−0.28 ± 0.96		−0.43 ± 0.65		−0.41 ± 0.81		−0.29 ± 1.07		−0.19 ± 0.88				ns
Ethnic Concentration (Mean ± SD)	1.44 ± 1.37		1.68 ± 1.63		1.81 ± 1.75		1.73 ± 1.47		1.07 ± 1.34		1.07 ± 1.27		F = 11.28	5, 600	<0.001

### Ethnicity

The projected sample size of 150 clients in the White North American origin group was obtained. Due to there being fewer than 50 clients of a specific ethnic origin groups presenting in a given year, the final sample sizes for the remaining five ethnic origin groups were as follows: 137 East Asian, 91 South Asian, 99 Black African, 141 Black Caribbean and 147 White European.

### Diagnosis

Schizophrenia was most frequently diagnosed in this sample (47.6%), followed by schizoaffective disorder (14.4%), Psychosis NOS/NYD (22%), a combination of diagnoses of which included schizophrenia/schizoaffective disorder (8.4%), and all other diagnoses (7.7%) including: brief psychotic episode, schizophreniform disorder, brief psychotic episode, first episode psychosis and substance induced psychosis. There were no statistically significant associations between ethnicity and diagnosis (Χ^*2*^ = 25.243, *df* = 20, *p* = 0.192).

### Sociodemographic and Clinical Characteristics

Table 2 presents sociodemographic and clinical characteristics of the sample with relevant test statistics. The average age of the sample was 38.01 (*SD* = 14.13) with the Black African (*M* = 33.5, *SD* = 11.9) and South Asian (*M* = 35.7, *SD* = 11.9) origin groups being the youngest. 36.7% of the sample was female. The Black Caribbean (41.6%) and East Asian (46.7%) origin groups had the highest proportion of females to males. Symptom severity ranged from 0 to 12 with a mean of 4.82 (*SD* = 2.54) and was highest in the Black Caribbean origin group (5.5 ± 2.8). Across the sample at the neighbourhood level, the level of residential instability ranged from −1.59 – 3.94 with a mean of 0.99 (*SD* = 1.23), the level of material deprivation ranged from −1.96 – 6.59 with a mean of 3.36 (*SD* = 1.28), the level of dependency ranged from −2.18 – 5.09 with a mean of −2.97 (*SD* = 8.69), and the level of ethnic concentration ranged from −1.22 – 6.49, with a mean of 1.42 (*SD* = 1.47). Black Caribbean (*M =* 0.68, *SD* = 1.18) and Black African (*M* = 0.82, *SD* = 1.56) origin clients reside in areas with higher levels of material deprivation. Black Caribbean (*M =* 1.81, *SD* = 1.75) and Black African (*M =* 1.73, *SD* = 1.47 origin clients also reside in areas with higher levels of ethnic concentration.

### Involuntary Detention

In the logistic regression model 2 (Table 3) predicting involuntary presentation under the MHA, after adjusting for neighbourhood level factors, East Asian origin clients are 2.36 times (95% CI 1.23–4.52) and South Asian origin clients are 2.99 times (95% CI 1.45–6.17) more likely to present to the ED involuntarily, when compared to White North American origin clients. Increased level of risk (2.12 OR, 95% CI 1.39–3.21), followed by symptom severity (1.25 OR, 95% CI 1.15–1.35) also results in increased odds of involuntary presentation. At the neighbourhood level only residential instability increased the odds (1.23 OR, 95% CI 1.05–1.45) of involuntary detention.

**Table 3:** Predictors of involuntary detention under the Mental Health Act on ED presentation in a sample of clients with psychosis

Variable	Model 1 OR (95% CI)	*p*	Model 2 OR (95% CI)	*p*
East Asian	1.87 (1.06–3.30)	0.030	2.36 (1.23–4.52)	0.010
South Asian	2.49 (1.34–4.62)	0.004	2.99 (1.45–6.17)	0.003
Black African	2.20 (1.19–4.06)	0.011	1.59 (0.78–3.24)	0.198
Black Caribbean	1.63 (0.94–2.84)	0.084	1.27 (0.66–2.47)	0.476
White European	1.29 (0.73–2.27)	0.377	1.11 (0.58–2.10)	0.761
Age	1.03 (1.02–1.04)	<0.001	1.02 (1.01–1.04)	0.001
Female	1.16 (0.82–1.63)	0.412	1.01 (0.68–1.50)	0.956
Symptom severity	1.21 (1.13–1.29)	<0.001	1.25 (1.15–1.35)	<0.001
Risk	1.94 (1.35–2.78)	<0.001	2.12 (1.39–3.21)	<0.001
Neighbourhood				
Residential instability			1.23 (1.05–1.45)	0.013
Material Deprivation			1.18 (0.98–1.42)	0.080
Dependency			0.97 (0.77–1.23)	0.842
Ethnic concentration			0.91 (0.76–1.08)	0.281
Constant	0.03	<0.001	0.03	<0.001
Χ^2^	91.78	<0.001	89.62	<0.001
df	9		13	

Reference Categories: gender (male); ethnicity (White North American); risk (no risk)

Sample size: model 1 (*n* = 725); model 2 (*n* = 574)

### Negative Mode of Arrival

Table 4 presents the models for negative mode of arrival. In the unadjusted regression (model 1) both East Asian (2.12 OR, 95% CI 1.21–3.71) and Black African (2.00 OR, 95% CI 1.10–3.64) origin clients have increased odds of presenting to the ED by police or ambulance when compared to the White North American origin reference group. After adjusting for involuntary mental health status (model 2), the differences between ethnic origin groups are no longer significant; however females have decreased odds (0.62 OR, 95% CI 0.42–0.93) of a negative presentation to the ED. Adjusting for neighbourhood factors in model 3 the odds of presenting via a negative mode of arrival are 2.1 times greater for East Asians (95% CI 1.01–4.36). Neighbourhood level residential instability increases the odds of a negative mode of arrival. In all models, having a GP reduces the odds of presenting to the ED by police or ambulance. Overall, involuntary status under the mental health act is the strongest predictor of a negative mode of arrival.

**Table 4:** Predictors of mode of arrival to ED by police or emergency services in a sample of clients with psychosis

Variable	Model 1 OR (95% CI)	*p*	Model 2 OR (95% CI)	*p*	Model 3 OR (95% CI)	*p*
East Asian	2.12 (1.21–3.71)	0.008	1.79 (0.96–3.35)	0.068	2.10 (1.01–4.36)	0.047
South Asian	1.77 (0.95–3.28)	0.070	1.18 (0.59–2.39)	0.633	1.56 (0.68–3.58)	0.296
Black African	2.00 (1.10–3.64)	0.023	1.61 (0.82–3.18)	0.166	1.74 (0.79–3.82)	0.169
Black Caribbean	1.51 (0.88–2.62)	0.138	1.28 (0.69–2.39)	0.436	1.50 (0.71–3.14)	0.285
White European	1.63 (0.94–2.81)	0.080	1.53 (0.83–2.83)	0.172	1.78 (0.89–3.60)	0.104
Age	1.03 (1.02–1.04)	<0.001	1.02 (1.01–1.04)	0.003	1.01 (0.99–1.03)	0.085
Female	0.75 (0.53–1.07)	0.111	0.62 (0.42–0.93)	0.020	0.69 (0.44–1.07)	0.097
Symptom severity	1.15 (1.08–1.23)	<0.001	1.08 (0.99–1.16)	0.062	1.06 (0.97–1.17)	0.181
GP	0.54 (0.39–0.76)	<0.001	0.67 (0.46–0.99)	0.043	0.65 (0.42–0.99)	0.047
Involuntary			9.83 (6.46–14.06)	<0.001	8.27 (5.31–12.87)	<0.001
Neighbourhood						
Residential instability					1.35 (1.13–1.62)	0.001
Material Deprivation					1.20 (0.98–1.48)	0.078
Dependency					1.20 (0.93–1.54)	0.153
Ethnic concentration					0.85 (0.70–1.03)	0.109
Constant	0.08	<0.001	0.07	<0.001	0.08	<0.001
Χ^2^	71.95	<0.001	216.84	<0.001	178.84	<0.001
Df	9		10		14	

Reference Categories: ethnicity (White North American); gender (male); GP (no GP), Involuntary (Voluntary)

Sample size: model 1 & 2 (*n* = 712); model 3 (*n* = 565)

### Accompaniment by Family or Friends

Adjusting for neighbourhood factors (Table 5), South Asian origin clients are 60% less likely (0.40 OR, 95% CI 0.17–0.95) to present with a family member or a friend when compared to White North American origin clients. Being in a relationship has the greatest increased odds of family and friend accompaniment. Higher risk and being older decreases the odds of accompaniment. Having a GP increases the odds, while having a psychiatrist decreases the odds of family or friend accompaniment. At the neighbourhood level, higher levels of ethnic concentration increases the odds of this mode of arrival, while rising levels of residential instability and material deprivation decreases the odds.

**Table 5:** Predictors of family or friend accompaniment to the ED in a sample of clients with psychosis

Variable	Model 1 OR (95% CI)	*p*	Model 2 OR (95% CI)	*p*
East Asian	1.11 (0.60–2.03)	0.748	0.75 (0.36–1.54)	0.434
South Asian	0.82 (0.41–1.65)	0.576	0.40 (0.17–0.95)	0.038
Black African	0.99 (0.50–1.96)	0.985	0.72 (0.32–1.63)	0.431
Black Caribbean	0.75 (0.40–1.42)	0.380	0.63 (0.29–1.36)	0.242
White European	1.46 (0.81–2.61)	0.209	1.37 (0.70–2.66)	0.358
Age	0.96 (0.95–0.98)	<0.001	0.98 (0.96–0.99)	0.007
Female	1.09 (0.73–1.63)	0.675	1.28 (0.80–2.06)	0.302
Partner	2.68 (1.55–4.64)	0.367	2.50 (1.31–4.79)	0.142
Symptom severity	1.04 (0.96–1.12)	0.005	1.07 (0.98–1.18)	0.029
Risk	0.53 (0.34–0.84)	0.049	0.55 (0.33–0.93)	0.026
GP	1.74 (1.19–2.55)	<0.001	1.65 (1.05–2.59)	0.005
Psychiatrist	0.69 (0.47–1.00)	0.006	0.61 (0.39–0.94)	0.025
Neighbourhood				
Residential instability			0.56 (0.46–0.69)	<0.001
Material Deprivation			0.74 (0.59–0.94)	0.012
Dependency			0.83 (0.61–1.13)	0.233
Ethnic concentration			1.33 (1.10–1.61)	0.004
Constant	0.89	0.758	0.69	0.450
Χ^2^	59.04	<0.001	95.88	<0.001
df	12		16	

Reference Categories: ethnicity (White North American); gender (male); partner (no partner); risk (no risk); GP (no GP); Psychiatrist (no Psychiatrist)

Sample size: model 1 (*n* = 676); model 2 (*n* = 532)

## Discussion

Our study is the largest to date to examine the relationship between ethnicity and pathways to emergency psychiatric care in clients with psychosis in Canada. To our knowledge this is also the largest study to examine involuntary detention under the MHA in Canada among a number of ethnic groups. Unlike previous studies, this study focuses on clients who are in crisis and presenting for emergency psychiatric care. There are differences in how clients are accessing services during emergency situations. An association was found between involuntary detention under the MHA and clients of East Asian and South Asian origins. Our findings, regarding ethnicity are different from previous Canadian studies where those of African and Caribbean origins had the highest risk of a police or ambulance referral [5]. Although South Asian origin clients in the UK are reported to have increased rates of detention [4], this may be higher in Canada. Our findings may relate to contextual differences in health service configuration, demographics [28], the social position of ethnic minorities [29] and a more nuanced categorization of ethnicity.

Despite an overall lower incidence of psychosis in East Asian origin groups in Ontario [30] and commonly presenting with fewer psychotic symptoms [31], this study found an increased risk of a negative pathways to emergency psychiatric care for this group. This finding could be viewed in the context of underutilization of mental health services by both South and East Asian-Canadians [32] and East Asian-Americans [33]. It has been suggested that East Asian-Americans seek mental health services only as a last resort [34], which may result in more challenging crises requiring coercive measures. Language, stigma and other factors may also play an important role, but this would not explain the lower levels of symptoms reported in our study.

Toronto may be unique in that there are a variety of ethno-specific services available for East and South Asian origin clients with severe and persistent mental illness [28, 35, 36]. Accessing these community based services may reduce mental health crises and ED presentations. There are ethno-specific community based mental health teams linked to other Toronto hospitals. These may also promote presentation to other emergency departments of people within these ethnic origin groups. If patients who are linked with services are more likely to be admitted elsewhere then the relative high rates of involuntary admission for this emergency department could reflect a sample bias. However, a recent study across Ontario argues against this, showing poorer outcomes from psychosis for Asian origin groups in general [11]. It argues that we need to improve our services for Asian origin groups.

At the individual level, having a primary care physician is associated with decreased odds of a negative pathway, this finding is in keeping with the literature [16]. Having a primary care provider may be indicative of a larger social network and greater organization of social supports available, both of which are associated with decreased frequency of hospitalization in individuals with a severe and persistent mental illness [37]. At the neighbourhood level, residential instability was associated with a coercive presentation, while increasing ethnic concentration was associated with a positive mode of arrival. Although we found a similar trend of increasing social fragmentation associated with negative outcomes for clients [38], we did not find any evidence of an association between material dependency - an area level socioeconomic factor - with negative pathways as previously suggested [13]. Our ethnic concentration findings are similar to UK studies which find ethnic minority clients in areas where minority groups make up a larger proportion of the population are more likely to experience positive mental health outcomes [39]. Residential patterns of ethnic groups in Toronto may be different, particularly as ethnic enclaves are common place [40]. Our results suggest there are implications relating to the importance of both social networks at the individual level and social cohesion at the neighbourhood level as part of the explanatory factors driving pathways to care.

This study is limited by the fact it was not designed to explain why such relationships may exist. A potential source of bias is also present due to the requirement that diagnosis, ethnicity and all outcomes variables be completed in the charts of clients that were included in the study. This was required as there is an increased likelihood of further incomplete data that pertains to predictor variables. Although we reached a large sample size of clients, we did not reach our expected sample size in all of the ethnic groups, which limits the power of this study. Furthermore, this study was conducted using data from one psychiatric ED in downtown Toronto, studies should be done to examine whether the associations found are replicable in other settings city-wide, provincially and nationally.

Methodologically, there are inherent challenges in describing ethnicity and culture in research as membership in both is dynamic [41]. To address this challenge this study approaches this issue through greater specificity in categorization of ethnic group membership. Previous studies have lumped African and Caribbean clients into a single heterogeneous group [5]. This can be problematic when significant differences may exist between people of specific ethnic subgroups. For instance in Toronto, when compared to people of Caribbean origin, those of African origin are more likely to experience poverty, to have a lower level of educational attainment, to be a refugee and speak English as a second language [29]. Furthermore, although this study focused on differences at the group level, it is important to note that variability exists within groups at the individual level that were not studied. These are all factors that may be important in pathways to care and may have important implications for both future research and clinical care.

## Conclusions

In Toronto, East Asian and South Asian origin clients with psychosis are more likely to present to emergency psychiatric services under coercive circumstances - being detained involuntary under the MHA or arriving via police or ambulance - and South Asian origin clients are less likely to be accompanied by family and friends. This study takes into account both clinical and neighbourhood factors in a client’s presentation, and finds that residential instability is associated with negative pathways, while ethnic concentration is associated with positive pathways. The differences found between ethnic origin groups are different than what has previously been described in Canada and abroad. Overall, these findings suggest that ethnic minority communities in Toronto access resources differently during crisis and may have unique experiences regarding individual level social networks and community level social cohesion. Further qualitative and quantitative investigation is required to determine exactly how these factors influence pathways to mental health service use.

## Abbreviations

CTO: Community treatment order
ED: Emergency department
GP: General practitioner
MHA: Mental health act
